# Bis[benzyl *N*′-(3-phenyl­prop-2-enyl­idene)dithio­carbazato-κ^2^ *N*′,*S*]mercury(II)

**DOI:** 10.1107/S1600536812025901

**Published:** 2012-06-16

**Authors:** M. A. A. A. A. Islam, M. S. Reza, M. T. H. Tarafder, M. C. Sheikh, E. Zangrando

**Affiliations:** a Department of Chemistry, Rajshahi University of Engineering and Technology, Rajshahi 6204, Bangladesh; b Department of Chemistry, Rajshahi University, Rajshahi 6205, Bangladesh; c Department of Applied Chemistry, Faculty of Engineering, Toyama University, 3190 Gofuku, Toyama 930-8555, Japan; d Department of Chemical and Pharmaceutical Science, Via L. Giorgieri 1, 34127, Trieste, Italy

## Abstract

In the title compound, [Hg(C_17_H_15_N_2_S_2_)_2_], the Hg^II^ ion lies on a crystallographic twofold rotation axis giving a very distorted tetra­hedral coordination geometry best described as bis­phenoidal, being chelated by two deprotonated *N*,*S* Schiff base ligands through the azomethine nitro­gen and the thiol­ate sulfur donors. The dihedral angle between the two chelating ligand moieties is 79.75 (10)°. In the crystal, weak C—H⋯S inter­actions give rise to chains extending along the *c* axis.

## Related literature

For the structures of uncoordinated Schiff bases, see: Tarafder, Crouse *et al.* (2008 ▶); Tarafder, Islam *et al.* (2008 ▶). For the corresponding Zn^II^ complex, see: Fun *et al.* (2008 ▶). For the coordination behaviour of metal ions (Co, Ni, Cu, Zn, Cd, and Hg) with the cinnamaldehyde Schiff base of *S*-methyl­dithio­carbazate, see: Liu *et al.* (2009 ▶); Abram *et al.* (2006 ▶). For the bioactivity of transition metal complexes of similar Schiff base ligands, see: Chew *et al.* (2004 ▶); How *et al.* (2008 ▶); Maia *et al.* (2010 ▶).
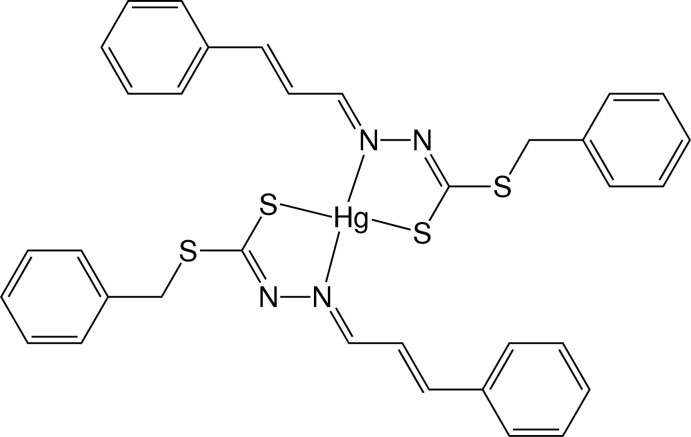


## Experimental

### 

#### Crystal data

[Hg(C_17_H_15_N_2_S_2_)_2_]
*M* *_r_* = 823.49
Orthorhombic, 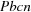

*a* = 36.3639 (6) Å
*b* = 10.11949 (10) Å
*c* = 8.77097 (10) Å
*V* = 3227.58 (7) Å^3^
*Z* = 4
Cu *K*α radiation
μ = 11.21 mm^−1^
*T* = 173 K
0.37 × 0.15 × 0.13 mm

#### Data collection

Rigaku R-AXIS RAPID CCD-detector diffractometer
Absorption correction: multi-scan (*ABSCOR*; Higashi, 1995 ▶) *T*_min_ = 0.194, *T*_max_ = 0.249
33943 measured reflections
2957 independent reflections
2829 reflections with *I* ^2^ > 2σ(*I* ^2^)
*R*_int_ = 0.113

#### Refinement

*R*[*F* ^2^ > 2σ(*F* ^2^)] = 0.038
*wR*(*F* ^2^) = 0.097
*S* = 1.20
2957 reflections
195 parameters
H-atom parameters constrained
Δρ_max_ = 1.76 e Å^−3^
Δρ_min_ = −2.04 e Å^−3^

### 

Data collection: *RAPID-AUTO* (Rigaku, 1995 ▶); cell refinement: *RAPID-AUTO*; data reduction: *RAPID-AUTO*; program(s) used to solve structure: *SHELXS97* (Sheldrick, 2008 ▶); program(s) used to refine structure: *SHELXL97* (Sheldrick, 2008 ▶); molecular graphics: *CrystalStructure* (Rigaku, 2010 ▶); software used to prepare material for publication: *publCIF* (Westrip, 2010 ▶). 

## Supplementary Material

Crystal structure: contains datablock(s) General, I. DOI: 10.1107/S1600536812025901/zs2213sup1.cif

Structure factors: contains datablock(s) I. DOI: 10.1107/S1600536812025901/zs2213Isup2.hkl

Additional supplementary materials:  crystallographic information; 3D view; checkCIF report

## Figures and Tables

**Table 1:** Selected bond lengths (Å)

Hg—S18	2.3668 (11)
Hg—N20	2.489 (3)

**Table 2:** Hydrogen-bond geometry (Å, °)

*D*—H⋯*A*	*D*—H	H⋯*A*	*D*⋯*A*	*D*—H⋯*A*
C6—H6⋯S16^i^	0.95	2.75	3.692 (4)	172

Symmetry code: (i) 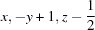.

**Fig. 1.**
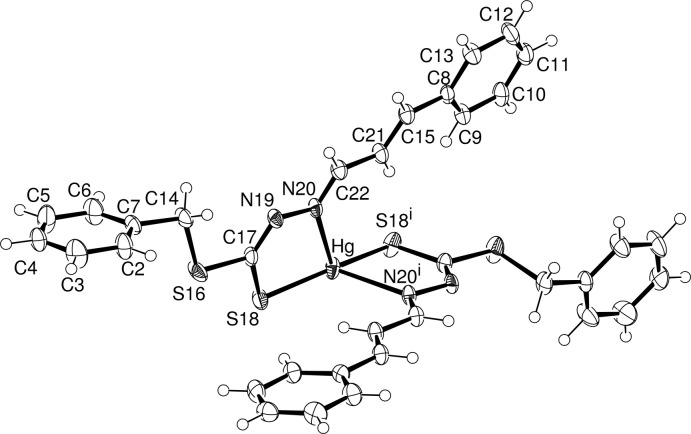
An ORTEP drawing (ellipsoids at the 50% probability level) of the title compound with atom labelling scheme of the independent moiety. For symmetry code: (i) -x + 1, y, -z + 3/2.

## supplementary crystallographic information

### Comment

In continuation of our interests in the chemistry of Schiff bases derived from *S*-benzyldithiocarbazate (Tarafder, Crouse *et al.*, 2008; Tarafder, Islam *et al.*, 2008) and on their metal complexes (Fun *et al.*, 2008) due to their intriguing coordination behaviour, physico-chemical properties, and potential biological activities, the title compound, [Hg(C_17_H_15_N_2_S_2_)_2_] (Fig. 1), was synthesized. In the structure, the Hg^II^ ion lies on a crystallographic twofold axis and has a very distorted tetrahedral coordination geometry best described as bisphenoidal, being chelated by two deprotonated benzyl *N'*-(3-phenylprop-2-enylidene)dithiocarbazate ligands through the azomethine nitrogen and the thiolate sulfur donors. The two chelating five-membered rings form a dihedral angle of 79.75 (10)°. The S–Hg–S' and N–Hg–N' bond angles, of 161.44 (4) and 92.57 (8)°, respectively, are closely comparable to those found in some Hg-thiosemicarbazone derivatives (Abram *et al.*, 2006). The S(18)—C(17) and the C(17)—N(19) bond distances [1.751 (4) and 1.302 (4) Å] are slightly longer and shorter in comparison with the values found in the free ligand [1.6696 (18) and 1.334 (2) Å, respectively (Tarafder, Crouse *et al.,* 2008)]. In the crystal packing the molecules are interconnected by weak C6—H6···S16 interactions [3.692 (4) Å] (Table 1), giving one-dimensional chain motifs extending along the *c* axis. The crystal is further stabilized by C–H···π interactions involving the phenyl ring of the 3-phenylprop-2-enylidene moiety.

### Experimental

The Schiff base, benzyl *N*'-(3-phenylprop-2-enylidene)hydrazinecarbodithioate was prepared following the literature method (Tarafder, Islam *et al.*, 2008). Mercury(II) chloride (0.068 g, 0.25 mmol) in absolute ethanol (20 ml) was added to a hot refluxing solution of the Schiff base (0.163 g, 0.5 mmol) also dissolved in hot absolute ethanol and the reflux was continued for 30 min. The yellow precipitate formed was filtered off, washed with hot ethanol and dried under vacuum over anhydrous CaCl_2_. Yield: 0.198 g (86%). 50 mg of the compound was dissolved in chloroform (15 ml) and allowed to stand at ambient temperature. Yellow microcrystals, obtained after 4 days, were redissolved in chloroform (15 ml) and mixed with toluene (5 ml) and again allowed to stand at room temperature. Yellow rectangular prism shaped single crystals (m.p. 472–473 K) suitable for X-ray analysis were formed after 7 days.

### Refinement

All H atoms were located geometrically and treated as riding atoms, with C—H = 0.95 Å for C(aromatic) and 0.99 Å, for C(methylene), with *U*_iso_(H) = 1.2*U*_eq_(C). The highest residual electron density peak (1.76 eÅ^-3^) is located at 0.60 Å from C1.

### Crystal data

**Table:**

[Hg(C_17_H_15_N_2_S_2_)_2_]	*D*_x_ = 1.695 Mg m^−^^3^
*M**_r_* = 823.49	Melting point = 472–473 K
Orthorhombic, *P**b**c**n*	Cu *K*α radiation, λ = 1.54187 Å
Hall symbol: -P 2n 2ab	Cell parameters from 33993 reflections
*a* = 36.3639 (6) Å	θ = 3.7–68.3°
*b* = 10.11949 (10) Å	µ = 11.21 mm^−^^1^
*c* = 8.77097 (10) Å	*T* = 173 K
*V* = 3227.58 (7) Å^3^	Prism, yellow
*Z* = 4	0.37 × 0.15 × 0.13 mm
*F*(000) = 1624.00	

### Data collection

**Table:**

Rigaku R-AXIS RAPID CCD-detector diffractometer	2829 reflections with *F*^2^ > 2.0σ(*F*^2^)
Detector resolution: 10.000 pixels mm^-1^	*R*_int_ = 0.113
ω scans	θ_max_ = 68.3°
Absorption correction: multi-scan (*ABSCOR*; Higashi, 1995)	*h* = −43→41
*T*_min_ = 0.194, *T*_max_ = 0.249	*k* = −11→12
33943 measured reflections	*l* = −10→10
2957 independent reflections	

### Refinement

**Table:**

Refinement on *F*^2^	Secondary atom site location: difference Fourier map
*R*[*F*^2^ > 2σ(*F*^2^)] = 0.038	Hydrogen site location: inferred from neighbouring sites
*wR*(*F*^2^) = 0.097	H-atom parameters constrained
*S* = 1.20	*w* = 1/[σ^2^(*F*_o_^2^) + (0.0485*P*)^2^ + 2.6351*P*] where *P* = (*F*_o_^2^ + 2*F*_c_^2^)/3
2957 reflections	(Δ/σ)_max_ = 0.001
195 parameters	Δρ_max_ = 1.76 e Å^−^^3^
0 restraints	Δρ_min_ = −2.04 e Å^−^^3^
Primary atom site location: structure-invariant direct methods	

### Special details

**Table:**

Geometry. ENTER SPECIAL DETAILS OF THE MOLECULAR GEOMETRY
Refinement. Refinement was performed using all reflections. The weighted *R*-factor (*wR*) and goodness of fit (*S*) are based on *F*^2^. *R*-factor (gt) are based on *F*. The threshold expression of *F*^2^ > 2.0 σ(*F*^2^) is used only for calculating *R*-factor (gt).

### Fractional atomic coordinates and isotropic or equivalent isotropic displacement parameters (Å^2^)

**Table:**

	*x*	*y*	*z*	*U*_iso_*/*U*_eq_	
Hg	0.5000	0.469781 (18)	0.7500	0.02693 (12)	
S16	0.62936 (2)	0.39052 (10)	0.66996 (11)	0.0407 (3)	
S18	0.56373 (3)	0.50749 (11)	0.78335 (13)	0.0324 (2)	
N19	0.56567 (6)	0.2929 (3)	0.5827 (3)	0.0238 (6)	
N20	0.52759 (6)	0.2998 (3)	0.5798 (3)	0.0230 (5)	
C2	0.71088 (9)	0.2399 (4)	0.5613 (5)	0.0378 (8)	
C3	0.74786 (9)	0.2696 (4)	0.5437 (6)	0.0460 (10)	
C4	0.75868 (9)	0.3722 (4)	0.4538 (5)	0.0389 (9)	
C5	0.73268 (11)	0.4492 (5)	0.3839 (5)	0.0474 (10)	
C6	0.69574 (10)	0.4202 (4)	0.4017 (5)	0.0421 (9)	
C7	0.68432 (8)	0.3154 (3)	0.4901 (4)	0.0264 (7)	
C8	0.41564 (8)	0.1159 (3)	0.3608 (4)	0.0236 (6)	
C9	0.39145 (8)	0.2117 (3)	0.4171 (4)	0.0285 (7)	
C10	0.35438 (9)	0.2050 (4)	0.3860 (4)	0.0335 (8)	
C11	0.34047 (10)	0.1024 (4)	0.2978 (5)	0.0351 (8)	
C12	0.36386 (15)	0.0076 (7)	0.2403 (4)	0.0370 (11)	
C13	0.40136 (14)	0.0130 (5)	0.2717 (5)	0.0330 (10)	
C14	0.64395 (9)	0.2869 (4)	0.5117 (4)	0.0315 (7)	
C15	0.45518 (8)	0.1197 (3)	0.3903 (4)	0.0265 (7)	
C17	0.58149 (8)	0.3828 (3)	0.6662 (4)	0.0244 (7)	
C21	0.47292 (9)	0.2093 (3)	0.4763 (4)	0.0264 (7)	
C22	0.51223 (10)	0.2110 (3)	0.4954 (4)	0.0256 (7)	
H2	0.7038	0.1670	0.6229	0.0453*	
H3	0.7658	0.2179	0.5949	0.0552*	
H4	0.7841	0.3903	0.4396	0.0467*	
H5	0.7400	0.5225	0.3233	0.0568*	
H6	0.6779	0.4735	0.3520	0.0505*	
H9	0.4007	0.2822	0.4774	0.0342*	
H10	0.3383	0.2707	0.4249	0.0402*	
H11	0.3149	0.0976	0.2773	0.0421*	
H12	0.3544	−0.0617	0.1788	0.0444*	
H13	0.4173	−0.0533	0.2327	0.0396*	
H14A	0.6400	0.1924	0.5358	0.0378*	
H14B	0.6300	0.3090	0.4182	0.0378*	
H15	0.4697	0.0526	0.3444	0.0318*	
H21	0.4588	0.2748	0.5271	0.0317*	
H22	0.5270	0.1465	0.4457	0.0307*	

### Atomic displacement parameters (Å^2^)

**Table:**

	*U*^11^	*U*^22^	*U*^33^	*U*^12^	*U*^13^	*U*^23^
Hg	0.02006 (18)	0.02382 (18)	0.03692 (18)	0.0000	0.00999 (6)	0.0000
S16	0.0147 (4)	0.0603 (6)	0.0470 (5)	−0.0065 (4)	−0.0006 (4)	−0.0233 (5)
S18	0.0250 (5)	0.0302 (4)	0.0418 (5)	−0.0094 (4)	0.0089 (5)	−0.0114 (4)
N19	0.0120 (12)	0.0279 (13)	0.0317 (13)	0.0007 (10)	0.0001 (10)	−0.0032 (11)
N20	0.0127 (12)	0.0237 (12)	0.0325 (13)	−0.0007 (10)	0.0023 (10)	−0.0015 (11)
C2	0.0201 (17)	0.0394 (19)	0.054 (3)	−0.0006 (14)	−0.0029 (16)	0.0114 (16)
C3	0.019 (2)	0.052 (3)	0.067 (3)	0.0085 (16)	−0.0066 (16)	0.0087 (19)
C4	0.0150 (17)	0.056 (3)	0.046 (2)	−0.0057 (15)	0.0020 (14)	−0.0027 (17)
C5	0.031 (3)	0.054 (3)	0.057 (3)	−0.0087 (18)	0.0024 (18)	0.018 (2)
C6	0.0227 (18)	0.049 (3)	0.055 (3)	0.0022 (16)	−0.0089 (16)	0.0142 (18)
C7	0.0141 (15)	0.0306 (15)	0.0345 (16)	−0.0000 (12)	−0.0018 (12)	−0.0061 (14)
C8	0.0174 (15)	0.0259 (15)	0.0274 (15)	−0.0047 (12)	0.0002 (12)	0.0032 (12)
C9	0.0211 (16)	0.0286 (16)	0.0359 (17)	−0.0043 (13)	0.0007 (13)	0.0000 (13)
C10	0.0176 (16)	0.0381 (18)	0.0449 (19)	−0.0011 (13)	0.0028 (14)	0.0050 (15)
C11	0.0217 (18)	0.047 (2)	0.0365 (18)	−0.0069 (15)	−0.0034 (16)	0.0086 (17)
C12	0.027 (3)	0.040 (3)	0.045 (3)	−0.012 (3)	−0.0067 (14)	−0.0019 (14)
C13	0.025 (3)	0.033 (3)	0.041 (2)	−0.0034 (18)	−0.0018 (16)	−0.0022 (15)
C14	0.0144 (16)	0.0412 (17)	0.0388 (18)	−0.0012 (13)	−0.0021 (13)	−0.0102 (16)
C15	0.0185 (15)	0.0268 (15)	0.0341 (16)	−0.0007 (12)	0.0022 (13)	−0.0007 (13)
C17	0.0148 (15)	0.0286 (16)	0.0296 (15)	−0.0026 (12)	0.0041 (12)	−0.0005 (12)
C21	0.0159 (17)	0.0281 (15)	0.0352 (16)	−0.0004 (12)	0.0000 (13)	−0.0021 (13)
C22	0.0190 (17)	0.0246 (14)	0.0332 (17)	0.0009 (12)	0.0019 (14)	−0.0017 (14)

### Geometric parameters (Å, º)

**Table:**

Hg—S18	2.3668 (11)	C10—C11	1.390 (6)
Hg—S18^i^	2.3668 (11)	C11—C12	1.378 (7)
Hg—N20	2.489 (3)	C12—C13	1.392 (8)
Hg—N20^i^	2.489 (3)	C15—C21	1.344 (5)
S16—C14	1.819 (4)	C21—C22	1.439 (5)
S16—C17	1.743 (3)	C2—H2	0.950
S18—C17	1.751 (4)	C3—H3	0.950
N19—N20	1.387 (3)	C4—H4	0.950
N19—C17	1.302 (4)	C5—H5	0.950
N20—C22	1.291 (4)	C6—H6	0.950
C2—C3	1.387 (5)	C9—H9	0.950
C2—C7	1.381 (5)	C10—H10	0.950
C3—C4	1.362 (6)	C11—H11	0.950
C4—C5	1.370 (6)	C12—H12	0.950
C5—C6	1.384 (6)	C13—H13	0.950
C6—C7	1.378 (5)	C14—H14A	0.990
C7—C14	1.508 (5)	C14—H14B	0.990
C8—C9	1.399 (5)	C15—H15	0.950
C8—C13	1.402 (6)	C21—H21	0.950
C8—C15	1.461 (5)	C22—H22	0.950
C9—C10	1.377 (5)		
			
S18—Hg—S18^i^	161.44 (4)	C15—C21—C22	123.4 (3)
S18—Hg—N20	77.93 (6)	N20—C22—C21	120.3 (3)
S18—Hg—N20^i^	115.59 (6)	C3—C2—H2	119.735
S18^i^—Hg—N20	115.59 (6)	C7—C2—H2	119.740
S18^i^—Hg—N20^i^	77.93 (6)	C2—C3—H3	119.689
N20—Hg—N20^i^	92.57 (8)	C4—C3—H3	119.649
C14—S16—C17	104.53 (15)	C3—C4—H4	120.216
Hg—S18—C17	99.93 (11)	C5—C4—H4	120.214
N20—N19—C17	114.6 (3)	C4—C5—H5	120.049
Hg—N20—N19	115.23 (17)	C6—C5—H5	120.064
Hg—N20—C22	130.6 (2)	C5—C6—H6	119.331
N19—N20—C22	114.1 (3)	C7—C6—H6	119.352
C3—C2—C7	120.5 (4)	C8—C9—H9	119.641
C2—C3—C4	120.7 (4)	C10—C9—H9	119.620
C3—C4—C5	119.6 (4)	C9—C10—H10	119.893
C4—C5—C6	119.9 (4)	C11—C10—H10	119.869
C5—C6—C7	121.3 (4)	C10—C11—H11	120.030
C2—C7—C6	118.0 (3)	C12—C11—H11	120.023
C2—C7—C14	121.2 (3)	C11—C12—H12	119.828
C6—C7—C14	120.7 (3)	C13—C12—H12	119.847
C9—C8—C13	118.6 (4)	C8—C13—H13	119.940
C9—C8—C15	122.5 (3)	C12—C13—H13	119.916
C13—C8—C15	118.9 (4)	S16—C14—H14A	110.606
C8—C9—C10	120.7 (3)	S16—C14—H14B	110.610
C9—C10—C11	120.2 (4)	C7—C14—H14A	110.602
C10—C11—C12	119.9 (4)	C7—C14—H14B	110.598
C11—C12—C13	120.3 (5)	H14A—C14—H14B	108.752
C8—C13—C12	120.1 (5)	C8—C15—H15	116.950
S16—C14—C7	105.7 (3)	C21—C15—H15	116.938
C8—C15—C21	126.1 (3)	C15—C21—H21	118.300
S16—C17—S18	108.96 (17)	C22—C21—H21	118.323
S16—C17—N19	118.9 (3)	N20—C22—H22	119.829
S18—C17—N19	132.1 (3)	C21—C22—H22	119.850
			
S18—Hg—N20—N19	3.69 (13)	Hg—N20—C22—C21	7.3 (4)
S18—Hg—N20—C22	178.9 (2)	N19—N20—C22—C21	−177.4 (3)
N20—Hg—S18—C17	−1.67 (7)	C3—C2—C7—C6	0.0 (6)
S18—Hg—N20^i^—N19^i^	170.22 (12)	C3—C2—C7—C14	−178.2 (4)
S18—Hg—N20^i^—C22^i^	−14.5 (3)	C7—C2—C3—C4	−1.2 (7)
N20^i^—Hg—S18—C17	85.38 (8)	C2—C3—C4—C5	2.0 (7)
S18^i^—Hg—N20—N19	170.22 (12)	C3—C4—C5—C6	−1.8 (6)
S18^i^—Hg—N20—C22	−14.5 (3)	C4—C5—C6—C7	0.6 (6)
N20—Hg—S18^i^—C17^i^	85.38 (8)	C5—C6—C7—C2	0.2 (6)
S18^i^—Hg—N20^i^—N19^i^	3.69 (13)	C5—C6—C7—C14	178.4 (4)
S18^i^—Hg—N20^i^—C22^i^	178.9 (2)	C2—C7—C14—S16	92.6 (4)
N20^i^—Hg—S18^i^—C17^i^	−1.67 (7)	C6—C7—C14—S16	−85.5 (4)
N20—Hg—N20^i^—N19^i^	−111.94 (15)	C9—C8—C13—C12	0.2 (5)
N20—Hg—N20^i^—C22^i^	63.3 (2)	C13—C8—C9—C10	0.2 (5)
N20^i^—Hg—N20—N19	−111.94 (15)	C9—C8—C15—C21	2.4 (5)
N20^i^—Hg—N20—C22	63.3 (2)	C15—C8—C9—C10	179.6 (3)
C14—S16—C17—S18	−166.34 (16)	C13—C8—C15—C21	−178.1 (3)
C14—S16—C17—N19	13.3 (3)	C15—C8—C13—C12	−179.3 (3)
C17—S16—C14—C7	167.73 (17)	C8—C9—C10—C11	−0.0 (5)
Hg—S18—C17—S16	179.53 (13)	C9—C10—C11—C12	−0.5 (6)
Hg—S18—C17—N19	−0.0 (3)	C10—C11—C12—C13	0.9 (6)
N20—N19—C17—S16	−176.2 (2)	C11—C12—C13—C8	−0.8 (7)
N20—N19—C17—S18	3.3 (5)	C8—C15—C21—C22	−177.1 (3)
C17—N19—N20—Hg	−4.6 (3)	C15—C21—C22—N20	−180.0 (3)
C17—N19—N20—C22	179.4 (3)		

Symmetry code: (i) −*x*+1, *y*, −*z*+3/2.

### Hydrogen-bond geometry (Å, º)

**Table:**

*D*—H···*A*	*D*—H	H···*A*	*D*···*A*	*D*—H···*A*
C6—H6···S16^ii^	0.95	2.75	3.692 (4)	172

Symmetry code: (ii) *x*, −*y*+1, *z*−1/2.
